# Color stability of aligner materials on exposure to indigenous food products: An in-vitro study

**DOI:** 10.34172/joddd.2022.035

**Published:** 2022-12-30

**Authors:** Priyanka Venkatasubramanian, Mary Sanjana Jerome, Lokamithra Ragunanthanan, Uma Maheshwari, Devaki Vijayalakshmi

**Affiliations:** Department of Orthodontics and Dentofacial Orthopaedics, Meenakshi Ammal Dental College and Hospital, Meenakshi Academy of Higher Education & Research, Mylapore, Chennai, India

**Keywords:** Aligner materials, Color stability, Food products, Indian, Indigenous products

## Abstract

**Background.:**

In the recent day, there has been an exponential growth in the usage of clear aligners for orthodontic treatment. As with any removable appliance, the compliance of patients to remove it during ingestion of food is, at times, poor. Thus, the stability of the clear aligner to be "clear" becomes questionable. This in-vitro study examined how the clear aligners changed colour on exposure to various indigenous food products used in everyday life.

**Methods.:**

Aligners from 5 different companies (K Line, Clearbite Aligners, The Aligner Company, iAligners and MaxDent CA Digital) were exposed for 12 hours and 24 hours to various indigenous substances (tea, green tea, coffee, turmeric, saffron and Kashmiri red chili powder) and a control solution (distilled water) in-vitro. The color change was assessed with the help of VITA Easyshade compact colorimeter based on Commission Internationale de I’Eclairage L*a*b* color system. Values were then modified to NBS units for clinical relevance.

**Results.:**

The hue of the transparent aligners was noticed to change in a statistically meaningful way when exposed to turmeric, saffron, Kashmiri red chili powder and coffee in decreasing order and mild color change in tea and green tea at both 12 hours and 24 hours intervals.

**Conclusion.:**

Aligners are prone to color change when exposed to indigenous foods that contain staining properties.

## Introduction

In this modern era, various orthodontic treatment modalities are influenced by aesthetic considerations associated with social perception.^1^ Patients seeking orthodontic treatment, both youngsters and adults, demand for a bracket free, aesthetic appliance.^2^ Thus, orthodontics has further evolved from aesthetic ceramic brackets or lingual appliances to clear aligners which not only has an increased comfort level but also aesthetic appealing, making it the most preferred choice of appliance, especially by the adults^3^ in turn increasing its demand. This increased desire for aligners has made them undergo various stages of development and modifications. The 1^st^ generation of aligners being solely reliant on the forces produced by the thermoplastic trays, the 2^nd^ generation having attachments for various movements like intrusion, extrusion, rotation and so on and the recent advancement includes the placement of these attachments using a software as part of the 3^rd^ generation.^4^

Clear aligner treatment changes the position of each tooth with the usage of a series of custom-made trays wherein each tray contributes to a limited amount of tooth movement. A set of aligner trays are worn for 2 weeks after which it is replaced by the next set, provided that the prescribed amount of tooth movement by that tray has been achieved successfully. If not, the individual must continue with the same tray.^4^

Generally, the thickness of the material used is 0.75mm, which over time reduces by 0.05mm with no change in the force delivery system.^5^ They are made using transparent thermoplastic materials like poly-urethane (PU), polyethylene terephthalate (PET), polyethylene terephthalate glycol (PETG), and polyvinyl chloride (PVC).^6,7^

Various studies focusing on mechanical properties and biomechanics^8^ of individual tooth movements in clear aligner treatment have been conducted. However, only a few investigations have been done on the colour and aesthetic stability of clear aligner materials. Ideally, from an aesthetic perspective, the color stability of each of these aligners should be maintained at least for the period of aligner wear.^9^ Factors like food substances, carbonated drinks, mouth rinses, and ultraviolet radiation greatly influence the color stability of various dental materials.^10^ Moreover, it is advisable to remove the aligners in course of food and beverages intake. But it has been stated in various studies that the compliance regarding removable appliance is insufficient and patients often continue wearing them during consumption of food.^11^ Hence, the pigments from the staining agents accumulate and results in discoloration and unaesthetic appearance of the aligners for such patients.^12^ Therefore, this raises a clinical concern and the need for investigation of the commonly used aligners and their color stability, which will give both the patient and the orthodontist, an idea of the clinical aesthetic considerations and the instructions that are needed to be followed with the use of the aligners.

 The current study’s goal was to assess in-vitro how clear aligners’ colour changes in response to being exposed to diverse indigenous food products.

## Methods

###  Aligner Material Collection

 Companies that manufacture clear aligners were contacted for study samples and explained about the research methodology. Of which 5 companies, namely, K Line (K Line Europe, Dusseldorf, Germany), Clearbite aligners (JJ Orthodontics, Kerala), The aligner company (Gujarat), iAligners (Delhi), MaxDent CA digital (Scheu-Dental, Germany) were willing to provide the required samples. A total of 7 maxillary aligners from each company were selected for the study.

Six agents, namely tea (Brooke bond), green tea (Lipton tea bag), coffee (Bru), turmeric powder (Sakthi masala), saffron (Patanjali), Kashmiri red chili powder (Sakthi masala) and as a control, distilled water was used. (Table 1)

**Table 1:** Staining agents

**Groups**	**Distribution of solutions**	
Group I	150 mL of distilled water	
Group II	1.5 g of Brooke Bond tea	150 mL of distilled water
Group III	1.5 g of Lipton green tea	150 mL of distilled water
Group IV	1.5 g of Bru coffee	150 mL of distilled water
Group V	1.5 g of Sakthi masala turmeric powder	150 mL of distilled water
Group VI	1.5 g of Patanjali saffron	150 mL of distilled water
Group VII	1.5 g of Sakthi masala Kashmiri red chili powder	150 mL of distilled water

###  Preparation

**Figure 1:**
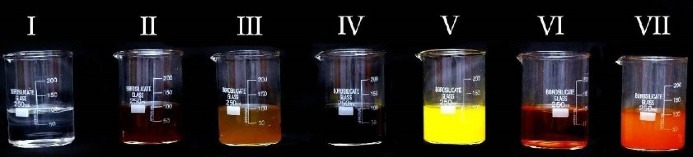
The preparation of the solutions

Each solution was prepared by mixing 1.5 g of the agent with 150 mL of boiling distilled water. The solution was cooled to 37°C (room temperature), subsequently was filtered out using a filter paper to eliminate any residues, thus obtaining a homogenous solution. The solution was constantly stirred every 30 minutes so that no sediments were being formed. Each solution was poured in separate containers (Figure 1).

###  Blinding

 A double blinding was done. The aligners from 5 different companies were numbered 1 to 5 by investigator 1 and the jars which contained the 7 different prepared solutions were labelled I to VII. Each aligner was taken from the solution by investigator 1 and was given to investigator 2 who assessed the measurement of color change. The values from investigator 2 were given to investigator 3 to derive at the results.

###  Reliability

 Two randomly selected aligners from each group of solutions were taken to examine the inter and intra-observer reliability. This evaluation process was repeated on two separate occasions by 2 investigators (investigator 2 and 3); there was an interval of one hour between the examination sessions, during which the aligners were preserved in dry and dark conditions to ensure no additional color change. Further, the interclass correlation coefficient (ICC) was calculated.

###  Procedure

 One aligner from each company was immersed in each of the 7 solutions. A tooth-shaped model of the right maxillary central incisor crown was made using packable composite cement of shade A2 (3M company) since this shade was considered the most common among the Indian population. This was done so that it acted as a background reference for color evaluation of the aligner material.

**Figure 2:**
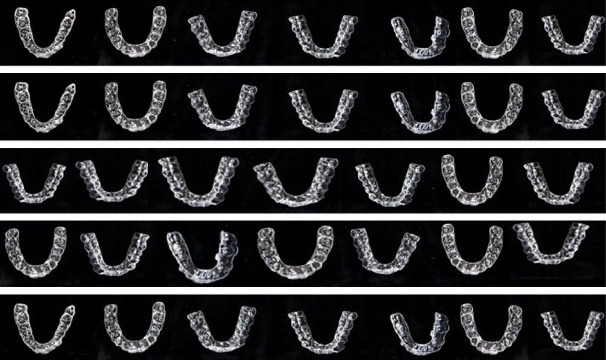
The aligners before immersion into the solution

Before placement into the solutions, the tooth model was placed into the aligner at its respective position for evaluation (T_0_) (Figure 2), following which the tooth model was removed and the aligners were immersed into the different solutions. After a period of 12 hours of immersion, the aligners were washed under running tap water and then dried with tissue paper. The tooth model was again placed back at its respective position for evaluation (T_1_) using the colorimeter. The aligners were then replaced into their respective solutions to repeat the procedure after 24 hours (T_2_).

###  Color change evaluation

A standard VITA Easyshade compact colorimeter was used to check the change in color, which was evaluated at 3 intervals. T_0_: before immersing into the solution, T_1_:12 hours after being immersed and T_2_: 24 hours after being immersed in the solution. All the measurements were done in the same room with a standardized light source.

The change in color was measured in accordance with the Commission Internationale de I’Eclairage L*a*b* color system. The color parameter L* represents the lightness ( + is lighter, - is darker), a*is the red/green coordinate ( + is redder, - is greener), and b* is the yellow/blue coordinate ( + is yellower, - is bluer). The total color change (ΔE*) [ΔE*=[(ΔL)2 + (Δa)2 + (Δb) 2] ^½^] value between T0 and T1 was calculated using the formula as (ΔE_T0-T1_)^2^ = (L*_T1_ – L*_T0_)^2^ + (a*_T1_ – a*_T0_)^2^ + (b*_T1_ – b*_T0_)^1/2^, and was similarly done between T_2 _– T_0_ and T_2_ – T_1_.

###  Color change rating

Color change rating was done with the help of the National Bureau of Standards System (NBS) of expressing color difference^13^ (Table 2).The ΔE value was converted into NBS units with the formula NBS = ΔE × 0.92 to relate the magnitude of color change to clinical relevance standard.^14^

**Table 2:** National Bureau of Standards ratings

**National bureau of standard ratings**	**Description of color change**
0.1–0.5	Trace: Extremely slight change
0.5–1.5	Slight: Slight change
1.5–3.0	Noticeable: Perceivable
3.0–6.0	Appreciable: Marked changes
6.0–12.0	Much: Extremely marked change
12.0 or more	Very much: Change to other color

###  Statistical analysis

Descriptive statistical analysis for each group was always applied after ΔE values were calculated using the above-mentioned formula. Kruskal-Wallis test revealed a significant result and hence, Turkey’s post hoc analysis (Mann-Whitney U tests) was performed to check for differences in color stability between groups and at different time periods. *P* value < 0.05 was considered as statistically significant. Friedman’s test was employed for the intra-group analysis to categorize the highly staining agent.

Qualitative evaluation was further done: the measured ΔE value was then converted into NBS value, which is presented in Table 3.

**Table 3:** NBS rating

**Groups**		**N**	**Mean**	**Standard deviation**	**NBS rating**	**Description of color change**
T0	1	5	0.0217	0.04857	0.01	Trace
	2	5	0.0000	0.00000	0.00	Trace
	3	5	0.0000	0.00000	0.00	Trace
	4	5	0.0217	0.04857	0.01	Trace
	5	5	0.0217	0.04857	0.01	Trace
	6	5	0.0217	0.04857	0.01	Trace
	7	5	0.0000	0.00000	0.00	Trace
T1	1	5	0.0434	0.05948	0.03	Trace
	2	5	0.6521	0.15373	0.59	Slight
	3	5	0.2825	0.12394	0.25	Trace
	4	5	0.6738	0.09094	0.56	Slight
	5	5	16.2826	0.17861	14.97	Appreciable
	6	5	15.04	0.94	13.83	Appreciable
	7	5	3.2391	0.82564	2.97	Noticeable
T2	1	5	0.0435	0.09718	0.04	Trace
	2	5	1.2608	0.12394	1.15	Slight
	3	5	0.5651	0.09094	0.51	Slight
	4	5	1.7172	0.09066	1.57	Noticeable
	5	5	16.9348	0.23566	15.57	Appreciable
	6	5	15.4347	0.81701	14.19	Appreciable
	7	5	7.6514	0.14541	7.03	Appreciable

## Results

The ICC values were calculated to determine the reliability of measurements. The ICC values ranged between 0.92 to 0.95 indicating a high level of agreement between the measurements (Table 4).

**Table 4:** Interclass correlation coefficient

	**Coefficient** **(Mean)**	**Standard deviation**	**t**	***P*** **>t**	**95% Confidence interval**	
Percent agreement	0.9268	0.0424	21.23	0.000	0.92	0.95
Conger’s/Cohen’s Kappa	0.9285	0.0464	20.35	0.000	0.9156	0.9448

**Figure 3:**
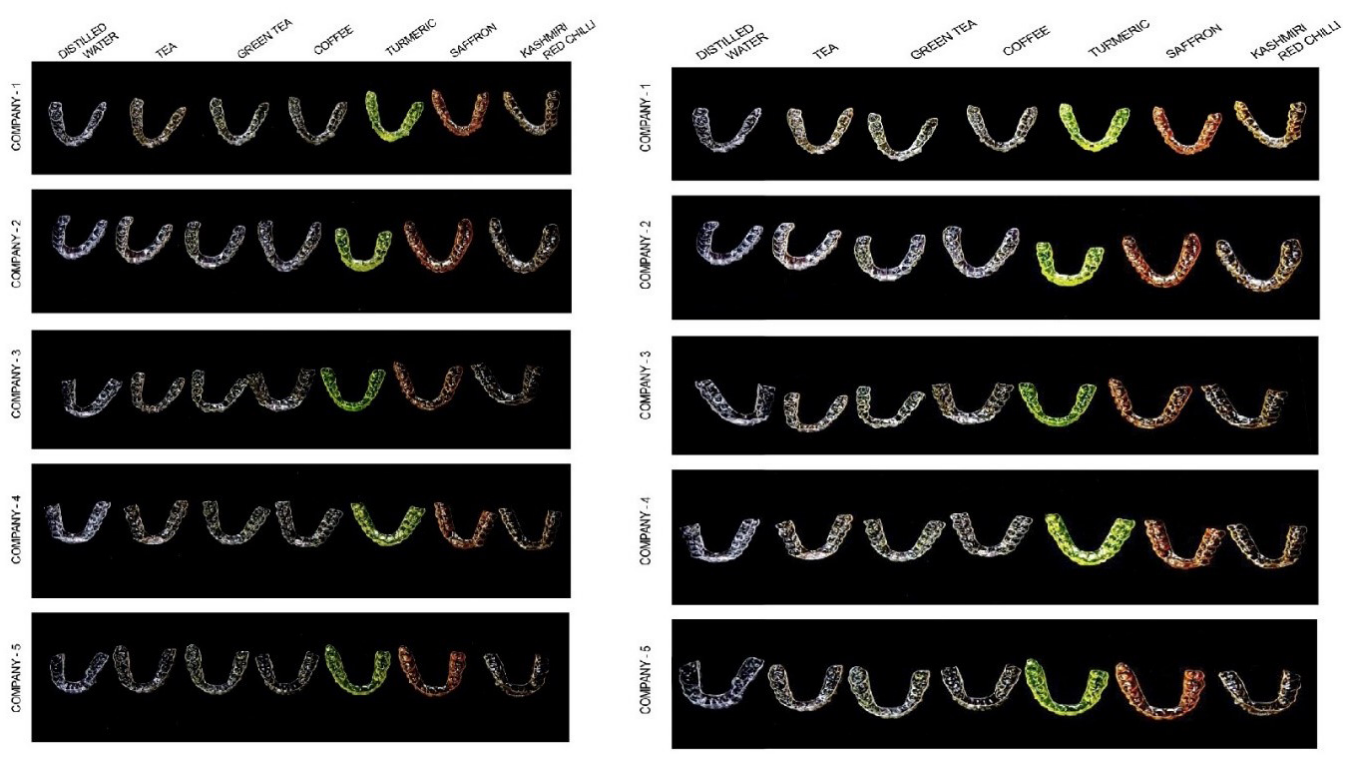
Photographs of the aligners after immersion (12 and 24 hours) in distilled water, tea, green tea, coffee, turmeric, Kashmiri chili powder and saffron

Photographs of the aligners before and after immersion (12 and 24 hours) in distilled water, tea, green tea, coffee, turmeric, Kashmiri chili powder and saffron are shown in Figure 3. Visual inspection shows that all the aligners were transparent before immersion into the solution (Figure 2). At T0, all the aligners showed a non-significant difference (*P* = 0.36) and the NBS value for T0 was in the category of trace: extremely slight change, for all the aligners irrespective of the group or the company.

In the inter-group analysis, all the aligners showed statistically significant differences at T1 as compared to the values obtained at T0 and T2. (Table 5). Post-hoc analysis done using Mann Whitney U test (Table 6) revealed that the color change between each of the groups were statistically significant except between group 2 and 4 at T1, which was not statistically significant.

**Table 5:** Intergroup comparison of colour stability

**Groups**		**N**	**Mean**	**Standard deviation**	**95% Confidence interval for mean**		***P*** ** value**
					**Lower bound**	**Upper bound**	
T0	1	5	0.0217	0.04857	-0.0386	0.0820	0.36
	2	5	0.0000	0.00000	0.0000	0.0000	
	3	5	0.0000	0.00000	0.0000	0.0000	
	4	5	0.0217	0.04857	-0.0386	0.0820	
	5	5	0.0217	0.04857	-0.0386	0.0820	
	6	5	0.0217	0.04857	-0.0386	0.0820	
	7	5	0.0000	0.00000	0.0000	0.0000	
T1	1	5	0.0434	0.05948	-0.0304	0.1173	< 0.001
	2	5	0.6521	0.15373	0.4612	0.8430	
	3	5	0.2825	0.12394	0.1286	0.4364	
	4	5	0.6738	0.09094	0.5609	0.7868	
	5	5	16.2826	0.17861	16.0608	16.2100	
	6	5	15.04	0.94	13.87	105908.10	
	7	5	3.2391	0.82564	2.2139	4.2643	
T2	1	5	0.0435	0.09718	-0.0772	0.1641	0.002
	2	5	1.2608	0.12394	1.1069	1.4147	
	3	5	0.5651	0.09094	0.4522	0.6781	
	4	5	1.7172	0.09066	1.6046	1.8298	
	5	5	16.9348	0.23566	16.6422	17.2274	
	6	5	15.4347	0.81701	14.4203	16.4492	
	7	5	7.6514	0.14541	7.4709	7.8320	

**Table 6:** Intergroup comparison between T1 and T2

**Post hoc analysis – Mann-Whitney test**						
**T1**	**2**	**3**	**4**	**5**	**6**	**7**
1	0.007	0.013	0.008	0.008	0.008	0.008
2		0.008	0.66	0.008	0.008	0.008
3			0.008	0.009	0.008	0.008
4				0.008	0.008	0.009
5					0.032	0.009
6						0.008
**T2**	**2**	**3**	**4**	**5**	**6**	**7**
1	0.007	0.007	0.007	0.007	0.007	0.007
2		0.008	0.008	0.009	0.009	0.009
3			0.008	0.008	0.009	0.009
4				0.008	0.009	0.008
5					0.008	0.009
6						0.009

 At T1, there was only a trace color change in group 1 and 3, wherein group 3 had a statistically significant change when compared to group 1 and a non-significant slight color change in group 2 and 4. A noticeable color change in group 7 and an appreciable color change in group 5 and 6 were evident, wherein group 5 and 6 revealed a statistically significant difference.

At T2, the findings were changed as compared to T1 (Figure 3). There was trace color change found only in the control group, while all other groups had a definite statistically significant change in color. There was a slight change in group 3 and group 2, while a noticeable color change in group 4. In contrast to T1, at T2 there was a statistically significant change between the groups 2 and 4 as revealed by the Mann-Whitney U test (Table 6). There was an appreciable change in group 5, 6 and 7, which were all statistically significant.

In the intra group analysis (Table 7), only group 1 had a non-significant difference, while all other groups had a statistically significant difference between T0 and T1, T0-T2 and T1-T2.

**Table 7:** Intragroup analysis – Friedman test

**Groups**	**N**	**Baseline**		**T1**		**T2**		***P*** ** value**	**Baseline vs T1**	**Baseline vs T2**	**T1 vs T2**
		**Mean**	**Standard** **Deviation**	**Mean**	**Standard** **Deviation**	**Mean**	**Standard** **Deviation**				
1	5	0.0217	0.04857	0.0434	0.05948	0.0435	0.09718	0.610	0.317	0.317	0.655
2	5	0.0000	0.00000	0.6521	0.15373	1.2608	0.12394	0.007	0.039	0.042	0.041
3	5	0.0000	0.00000	0.2825	0.12394	0.5651	0.09094	0.007	0.042	0.041	0.038
4	5	0.0217	0.04857	0.6738	0.09094	1.7172	0.09066	0.007	0.041	0.042	0.041
5	5	0.0217	0.04857	16.2826	0.17861	16.9348	0.23566	0.007	0.042	0.042	0.042
6	5	0.0217	0.04857	15.04	0.94	15.4347	0.81701	0.007	0.043	0.043	0.042
7	5	0.0000	0.00000	3.2391	0.82564	7.6514	0.14541	0.007	0.043	0.042	0.043

## Discussion

With conventional orthodontic treatment, elastics, modules, ceramic brackets and e-chains are prone to color change over time.^15^ Though the aligners are generally “clear”, they tend to change color due to the pigments that are being absorbed from the food items by the constituents of aligner material. Hence, removing them during food consumption is a better and ideal way to maintain the aligners “clear”, which is often the guidelines issued by the manufacturers of these aligners.^16^ But with the fast-paced way of life, patients often restrain themselves from removing them while eating, due to rationales like, embarrassment of removing in the public, the sense of feeling unhygienic during reinsertion in a communal environment, the fear of misplacement or damaging while it’s not being worn. Exceptionally, companies like MaxDent CA digital, recommend patients to the usage of aligners with a soft diet, as they incorporate attachments which works better with functions like mastication. Hence, they provide 3 sets of varying texture of aligners (soft, medium & hard) per month, each for 10 days.^17^

 There are variety of ingredients which are found to stain the teeth and the oral appliances, like the pasta sauces which are inherently acidic, curry that has common spice which are often found in Indian food and other exotic dishes, beverage drinks like coffee, tea, wine, and sports drinks, berries and balsamic vinegar. Although these above-mentioned substances are commonly used in all cultures, it is worthwhile to identify the ethnic differences in food and its influence on oral appliances, especially the aligners. Spices like turmeric, saffron and other masalas which are a mandatory ingredient of any Indian cooking cause a major concern as they are used in day-to-day food preparations. Thus, in this study we sought to evaluate the extent to which these Indian exogenous colorants and spices stain the aligners when patients continue to wear the aligners during consumption of food, despite the manufacturer’s instructions.

In this study, turmeric powder, saffron powder and Kashmiri red chili powder were preferred to check the color stability of clear aligners, because these agents have the potential to color the food and are used as a vital ingredient in Indian cooking. Curcumin is the deep yellow coloring material found in turmeric and crochin is a water-soluble carotenoid that is responsible for the staining property of saffron. These ingredients are added in a minimal quantity as they not only impart color but also spice and flavor. Hence, these agents cannot be avoided as far as Indian cuisine is concerned.^18^ Hot beverages like coffee, tea, and green tea are some of the most commonly consumed non-alcoholic beverages among Indian population, of which coffee being the strongest staining agent.^19^ Tannin compounds present in tea and coffee causes the deep stain. Statistics show, on an annual per capita basis, Indians drink 15.6 cups of coffee.^20^ 1.5 g of the staining agents were taken and dissolved in 150 mL of distilled water that was boiled to 100°C so that the agents mix thoroughly. The solution was constantly stirred to achieve a homogenous solution.

 With regards to the sample collection, companies provided the needed gratis sample of maxillary and mandibular aligner trays through courier. For uniformity of the study, only the maxillary aligner trays were chosen as the majority of mandibular trays were damaged. Moreover, they were also requested to provide the details regarding the materials used in the manufacturing process of the same. Segregating them according to the material used was not done as the exact material composition was not known. Nevertheless, in our study we included different aligner companies so that a randomization of the aligners can be obtained in the sense of their variation in transparencies by different manufacturers.

Measurements of color change were recorded in a dark room without any natural light, under a standardized light source of 6500 K LED bulb as recommended for the usage of VITA Easyshade advance 4.0. It demonstrates reliable, repeatable values and is 93.75% accurate when used for in-vitro models.^21^

To mimic the intra-oral set up, the values were recorded with an incisor shaped tooth-model cured using 3M packable composite of shade A2 as a reference, placed at the central incisor slot of the clear aligners. Shade A2 was chosen as it corresponds to 2M2 VITA 3D shade, which is prevalent among the Indian population.^22^ In-vitro studies usually provide an initial estimate, which was calculated in a previous study,^23^ wherein, each cup of coffee is consumed for 15 minutes and on an average 3.2 cups per day. Thus, 12 hours and 24 hours of in-vitro immersion simulates 2 weeks and 4 weeks or monthly consumption of coffee.

**Figure 4:**
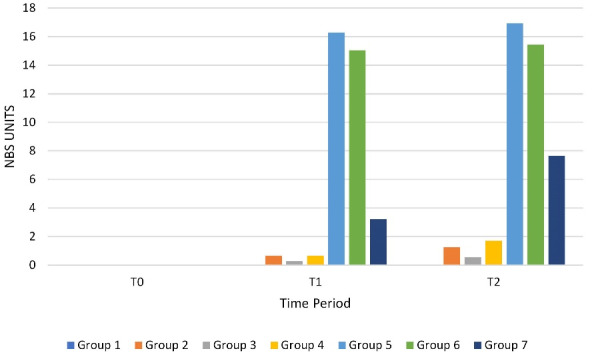
Marked staining with turmeric, followed by saffron, Kashmiri red chili powder, coffee, tea and green tea in descending order

Further, the change in color was measured in accordance to the Commission Internationale de I’Eclairage L*a*b* color system and derived at the National Bureau of Standards System of expressing color difference^19^ for T0, T1 and T2, so that the magnitude of color change can be related to clinical relevance standard (Figure 4).^14^

 In our study, before immersion, there was a trace (extremely slight) change of color observed for all the aligners. Thus, it can be concluded that all the aligners were of the same degree of transparency at the beginning of the study.

After 12 hours of immersion in the solution groups, all the aligners showed statistically significant differences when compared to T0. Inter group comparison at T1 showed significant color change amongst the solution groups, except between tea and coffee that showed a non-significant slight color change. There was only a trace color change in water and green tea, wherein the green tea group had a statistically significant change when compared to the control group. A noticeable color change in Kashmiri red chili powder and an appreciable color change in turmeric and saffron were seen, wherein turmeric group was statistically significant among them. Thus, we concluded that a marked staining was seen with turmeric, followed by saffron, Kashmiri red chili powder, coffee, tea and green tea in descending order (Figure 4).

Moreover, in our study, after 24 hours, the staining further intensified as compared to the 12 hours post immersion (Figure 3). There was a statistical significance between T2 and T1 and also among the aligners in different solutions. There was a slight change in green tea and tea, while a noticeable amount of change in coffee. There was an appreciable change in turmeric, saffron and Kashmiri red chili, which were all statistically significant. Thus, we concluded that there was an increased staining found with turmeric, which completely turned yellow, followed by saffron, Kashmiri red chili powder and coffee, tea having varying degrees of yellow to brown shades. The order of staining between the agents did not vary between 12 hours and 24 hours. No perceivable color changes were observed in any type of aligners after immersion in distilled water during both time periods.

In research conducted by Liu et al, polyurethane-based Invisalign was more prone to coffee stains followed by black tea and red wine at 12 hours and at 7 days of immersion.^16^ According to Um and Ruyter, coffee stains more than tea, due to the presence of yellow dye in coffee.^24^

 The Invisalign instructions for patients suggest that drinking coffee with the aligner in the mouth should be avoided.

Bernard et al^25^ concluded that coffee and red wine significantly stains the Invisalign compared to ClearCorrect^®^ and Minor Tooth Movement^®^ after 12 hours and 7 days. Black tea imparted a significant color change after 7 days on Invisalign, ClearCorrect^®^ and Minor Tooth Movement^®^.

With the increased preference for clear aligner therapy, there is a proportionate concern on the biocompatible properties of thermoplastic materials being used. With betterment in technology, there are various newer brands of thermoplastic sheets available, which are needed to be tested. Materials like PU, PET, and PETG are amorphous polymers exhibiting the property of transparency which are widely used in production of clear aligners with various other applications in the dental field.PU expresses favorable properties like ease of processing, chemical resistance, flexibility and elasticity.^26^ Fang et al reported that “Invisalign”, which is made of a modified polyurethane material, when used for over 2 weeks, resulted in delamination, voids and cracks. Whereas, transmission electron microscopy revealed no significant change in internal structure and edges at the end of 2 weeks of usage.^27^ PVC is highly recommended due to their excellent optical, chemical and physical characteristics.^28^ PETG is a non-crystalline amorphous co-polymer of PET with the advantage of dimensional stability, fatigue resistance and formability.^29^

The differences in the color changes of aligners may have been due to their different composition of polymer based materials, which could be susceptible to stains in different ways. PU materials exhibit a higher amount of water absorption as they contain a polar group (-NHCOO-) which is reactive with the hydrogen bonds of the hydrophilic pigments in solutions, than PVC and PETG in the intra-oral environment. On the other hand, the chemical group “-COO-” and “-C-O-C-” that are present in PVC and PETG have lesser polar potential.^16,30^ Among our study samples, iAligners (procure their thermoplastic sheets from Forestadent, Germany) and Clearbite aligners are polyurethane based, K-line and MaxDent CA digital aligners are PETG based, whereas TAC is a combination of PETG and PU base.

When aligners are stained, patients tend to cleanse them using diluted hydrogen peroxide/ solution of baking soda/ vinegar/ soap water/ commercially available products like Invisalign cleaning crystals^®^ that is part of the Invisalign kit, Retainer Bright^®^, Sonic Bright^®^, etc. Few dentists use the in – office type of cleansing: sonic cleaner or ultrasonic scalers to remove the stains. Bernard et al experimented and pointed out that with the use of Invisalign crystals and Retainer Brite tablets, only the tea stains that were present on the aligners exposed to tea over a period of 7 days, showed a positive result. On the contrary, cleansing of aligners stained due to coffee and red wine pigments with the same crystals and tablets gave unsatisfactory results with the presence of marked color change.^25^

###  Limitations

- Since it is an in-vitro study, exact oral environments like the presence of plaque or bacteria could not be incorporated and hence their role in color stability can be a future scope of study.
- As per company’s policy, detailed material composition could not be obtained, hence sample segregation based on composition could not be done.
- In this study, aligners were rinsed only at the end of 12 and 24 hours. Rinsing them at a frequent time interval, simulating the daily brushing and cleaning could have been included.
- Change of color with different temperatures of the solutions was not investigated.
- Variation in the quantity of the indigenous products was also not assessed.

## Conclusion

 Color stability of the aligners evaluated in this study revealed that aligners are more prone to pigmentation when exposed to indigenous products. Turmeric stains the most, followed by saffron, Kashmiri red chili, coffee, tea and green tea being the least. The staining is statistically significant with an increase in the exposure time to the products. New aligner materials which are more resistant to such staining must be improvised. Moreover, orthodontists must reinforce the fact that consumption of such indigenous products while wearing the aligners have a negative effect on aesthetics and transparency of the clear aligners, which loses its sole purpose of preference over conventional treatment modalities.

## Funding

 None.

## Ethics Approval

 The study proposal was approved by the Institutional Review Board (IRB) before commencement of the study (Ethical approval number: MADC/IRB-XXVII/2019/240).

## Competing Interests

 None.
